# The conformation of the nSrc specificity-determining loop in the Src SH3 domain is modulated by a WX conserved sequence motif found in SH3 domains

**DOI:** 10.3389/fmolb.2024.1487276

**Published:** 2024-12-03

**Authors:** Frederick Longshore-Neate, Caroline Ceravolo, Cole Masuga, Elise F. Tahti, Jadon M. Blount, Sarah N. Smith, Jeanine F. Amacher

**Affiliations:** Department of Chemistry, Western Washington University, Bellingham, WA, United States

**Keywords:** SH3 domains, protein-protein interactions, protein-peptide interactions, short linear motifs, specificity, signal transduction

## Abstract

Cellular signaling networks are modulated by multiple protein-protein interaction domains that coordinate extracellular inputs and processes to regulate cellular processes. Several of these domains recognize short linear motifs, or SLiMs, which are often highly conserved and are closely regulated. One such domain, the Src homology 3 (SH3) domain, typically recognizes proline-rich SLiMs and is one of the most abundant SLiM-binding domains in the human proteome. These domains are often described as quite *versatile*, and indeed, SH3 domains can bind ligands in opposite orientations dependent on target sequence. Furthermore, recent work has identified diverse modes of binding for SH3 domains and a wide variety of sequence motifs that are recognized by various domains. Specificity is often attributed to the RT and nSrc loops near the peptide-binding cleft in this domain family, particularly for Class I binding, which is defined as RT and nSrc loop interactions with the N-terminus of the ligand. Here, we used the Src and Abl SH3 domains as a model to further investigate the role of the RT and nSrc loops in SH3 specificity. We created chimeric domains with both the RT and nSrc loop sequences swapped between these SH3 domains, and used fluorescence anisotropy assays to test how relative binding affinities were affected for Src SH3- and Abl SH3-specific ligands. We also used Alphafold–Multimer to model our SH3:peptide complexes in combination with molecular dynamics simulations. We identified a position that contributes to the nSrc loop conformation in Src SH3, the amino acid immediately following a highly conserved Trp that creates a hydrophobic pocket critical for SH3 ligand recognition. We defined this as the WX motif, where X = Trp for Src and Cys for Abl. A broad importance of this position for modulating nSrc loop conformation in SH3 domains is suggested by analyses of previously deposited SH3 structures, multiple sequence alignment of SH3 domains in the human proteome, and our biochemical and computational data of mutant Src and Abl SH3 domains. Overall, our work uses experimental approaches and structural modeling to better understand specificity determinants in SH3 domains.

## Introduction

Short linear motif (SLiM) or peptide binding is a critical component of signal transduction pathways, often with several SLiM-binding domains in multiple proteins modulating the activity and regulation of the signaling cascade (Pawson and Nash, 2003). One such domain, the Src-homology 3 (SH3) domain, was first described in 1988 as a domain located on the proto-oncogene Src tyrosine kinase ((Mayer et al., 1988; Stahl et al., 1988)). It is now recognized that SH3 domains exist in all kingdoms of life and viruses, and there are over 300 SH3 domains in 200 proteins in the human proteome ((Mehrabipour et al., 2023; Teyra et al., 2017)). Although several noncanonical exceptions exist, SH3 domains are generally characterized as binding to proline-rich sequences, specifically containing a PXXP motif (where X = any amino acid), with affinities in the low-to-mid micromolar range [(Teyra et al., 2017; Pisabarro and Serrano, 1996)]. Many SH3 target sequences adopt a type II polyproline helix (PPII) structure, presenting a hydrophobic surface to which the SH3 domain recognizes and binds [(Lim et al., 1994; Mayer, 2001)].

**FIGURE 1:**
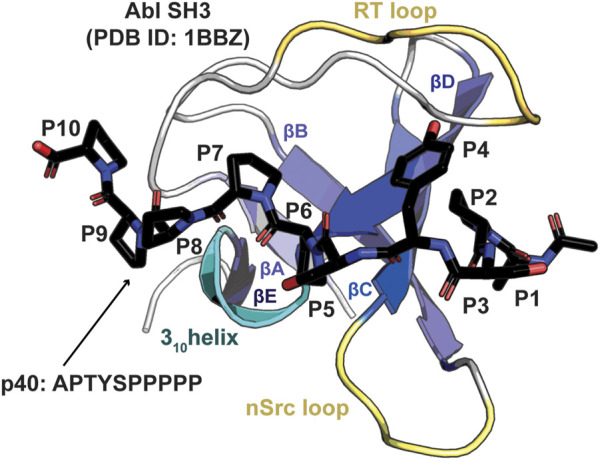
Structure of an SH3 domain. The Abl SH3 structure is shown as a cartoon bound to the high affinity p40 (APTYSPPPPP) peptide, which is shown in stick representation and colored by atom (C = black, O = red, N = blue), PDB ID: 1BBZ (Pisabarro et al., 1998). Conserved structural elements are highlighted and labeled, including the βA-βE strands, 3_10_ helix, and RT and nSrc loops. Peptide positions, P1-P10, are labeled.

SH3 domains share a general conserved structure, despite displaying a relatively large degree of plasticity in ligand binding. SH3 domains are typically about 60 amino acids in length, with a β-barrel fold, consisting of approximately 5 β-strands and a 3_10_ helix [(Mehrabipour et al., 2023; Kaneko et al., 2008a)] (Figure 1). Specificity in SH3 domain binding is determined by the nSrc, RT, and β4-α3_10_ loops (Mehrabipour et al., 2023). Interestingly, the same SH3 domain can bind ligands in opposite orientations, depending on whether it corresponds to a Class I/“plus” (consensus sequence: RXLPPXP, where X = any amino acid) or Class II/“minus” (XPPLPXR) target sequence [(Lim et al., 1994), (Feng et al., 1994; Kurochkina and Guha, 2013; Saksela and Permi, 2012)]. In the Class I orientation, N-terminal residues of the ligand interact directly with the RT and nSrc loops; in the Class II orientation, these regions interact with C-terminal residues of the ligand [(Lim et al., 1994; Feng et al., 1994)]. Thus, the same SH3 domain can bind ligands N- to C-terminal or *vice versa* depending on the target sequence.

The *Src module*, consisting of an SH3 domain, SH2 domain, and tyrosine kinase, is shared amongst several families of cytoplasmic tyrosine kinases (Shah et al., 2018). This includes Abl, a Src-related tyrosine kinase, which regulates actin (Colicelli, 2010). The kinase domains of Src and Abl are 46% identical, but these proteins are known to be differentially regulated [(Seeliger et al., 2007; Sicheri et al., 1997; Eck et al., 1994; Nagar et al., 2003)]. Furthermore, therapeutics that target the BCR-Abl fusion protein, which is the underlying cause of disease in approximately 95% of chronic myelogenous leukemia (CML) cases, e.g., imatinib, bind and inhibit Abl kinase, but do not bind Src kinase (Seeliger et al., 2007). There are also specificity differences in the SH3 domains of these proteins, which are 43% identical over 58 residues. A previous study of SH3 domain specificity investigated an initial ligand, derived from the 3BP1 protein (sequence: RAPTMPPPLPP), which bound the Abl and Fyn (a Src family kinase, the SH3 domains of Fyn and Src share 77% sequence identity) SH3 domains with similar 30 μM affinity [(Pisabarro and Serrano, 1996; Pisabarro et al., 1998)]. This sequence was then used as a template to design a selective and high affinity Abl SH3 ligand, termed p40 [(Pisabarro and Serrano, 1996; Pisabarro et al., 1998)]. The Class I p40 ligand (APTYSPPPPP) bound the Abl and Fyn SH3 domains with 0.4 μM and 470 μM affinity, respectively [(Pisabarro and Serrano, 1996; Pisabarro et al., 1998)].

Here, we aimed to better understand this result by expanding this investigation to Abl and Src SH3 specificity using chimeric proteins, Abl_Src_ and Src_Abl_, which swap both the RT and nSrc loop sequences from the other domain. We calculated binding affinities of these chimeric domains with the Class I p40 ligand, as well as a Class I Src-specific target sequence, LASRPLPLLP, termed PLLP (Sparks et al., 1996). We found that while the WT Src and Abl SH3 domains are specific for their ligands, the Src_Abl_ chimeric protein revealed relatively weak but similar binding to both peptide sequences. To visualize the tested SH3:ligand interactions, we used Alphafold–Multimer to model the complexes and analyze potential binding interfaces, in combination with molecular dynamics simulations for Src SH3 variants. We also tested single mutations of a WW (for Src SH3) and WC (for Abl SH3) sequence motif at the C-terminus of the nSrc loop in the wild-type and chimeric proteins, showing that these can modulate ligand affinity several fold. These WX positions in Src SH3, the amino acids W121 and W122, have previously been shown to be important for establishing a hydrophobic binding specificity pocket and modulating lipid binding [(Sipeki et al., 2021; Le Roux et al., 2019)]. Here, we argue that the W122 position, or X of the WX motif, additionally determines the conformation (and potentially the flexibility as well) of the nSrc loop. Taken together, our studies provide further insight into the role of these specificity-determining loops for the Src and Abl SH3 domains.

## Results

### Differing specificities of Src and Abl SH3 domains

**FIGURE 2:**
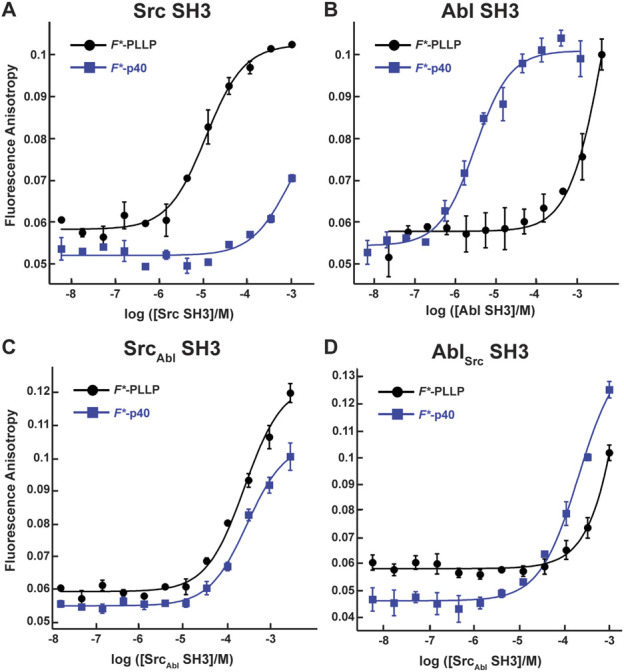
Binding affinities for F*-PLLP and p40 with Src, Abl, Src_Abl_, and Abl_Src_ SH3 domains. The binding curves for the Src **(A)**, Abl **(B)**, Src_Abl_ **(C)**, and Abl_Src_ **(D)** SH3 domains with the F*-PLLP (LASRPLPLLP) and F*-p40 (APTYSPPPPP) peptides. Averaged values and standard deviations are shown for each and calculated binding affinities are labeled and are in Table 1. Because of differing protein concentrations, the data in these graphs may not include all replicates used for binding affinity measurements.

To investigate the effect of RT and nSrc loop variation on the Src and Abl SH3 domains, we first wanted to confirm that these domains contain differing ligand specificities. We recombinantly expressed and purified the Src and Abl SH3 domains as SUMO-tagged fusion proteins, as described in the Materials and Methods. All sequences used in this work are in the **Supporting Information**. We then used fluorescence anisotropy experiments, as described previously and in the Materials and Methods, to test the binding affinities of each domain with two fluorescein (F*)-tagged peptides, one corresponding to the p40 sequence, F*-Ahx-APTYSPPPPP, termed F*-p40, and to a Src SH3 identified ligand, F*-Ahx-LASRPLPLLP, termed F*-PLLP (Figure 2; Table 1) [(Pisabarro and Serrano, 1996; Pisabarro et al., 1998; Sparks et al., 1996)]. For clarity, we will refer to peptide positions numerically from the N-terminus and include one-letter amino acid codes for identification, e.g., for p40, A1-P2-T3-etc.

**TABLE 1:** Relative binding affinities using fluorescence anisotropy for Src- and Abl-derived SH3 domains.

	*K*_d_ (μM)	
	F*-PLLP	F*-p40
Src SH3	9.7 ± 0.9	>1,000
Src_Abl_ SH3	140 ± 20	130 ± 40
W122C Src SH3	810 ± 160	>1,000
W122C Src_Abl_ SH3	>1,000	450 ± 190
Abl SH3	>1,000	3.1 ± 0.4
Abl_Src_ SH3	>1,000	210 ± 30
C100W Abl SH3	>1,000	55 ± 20

Consistent with previous work, our results indicated that while Src SH3 bound F*-PLLP with relatively high affinity, *K*_D_ = 9.7 ± 0.9 μM, it did not bind F*-p40, here defined as *K*_D_ > 1,000 μM (Figure 2A; Table 1). In contrast, Abl SH3 bound F*-p40 with *K*_D_ = 3.1 ± 0.4 μM, and F*-PLLP with *K*_D_ > 1,000 μM (Figure 2B; Table 1). Notably, our Abl SH3 affinity for F*-p40 differs by an order of magnitude as compared to the previously published value, 0.4 ± 0.1 μM (for reference, in this paper Fyn SH3 bound with *K*_D_ = 472 ± 55 μM) [(Pisabarro and Serrano, 1996; Pisabarro et al., 1998)]; however, we attribute these differences to the experimental methods used, variations in sequence, i.e., our peptides also include N-terminal fluorescein and Ahx linkers, and the use of Fyn SH3 as opposed to Src SH3, whose SH3 domains are 77% identical by sequence, as stated previously. Despite variation in absolute values, our results also indicated a difference of approximately three orders of magnitude for Abl SH3 binding to F*-p40 as compared to Src/Fyn SH3, indicating internal consistency with the previous results. Taken together, our results confirmed differing ligand specificities for the Abl and Src SH3 domains.

### Chimeric SH3 domains confer differences in ligand specificities

Previous reports indicated that the RT and nSrc loops of SH3 domains drive ligand specificity (Mehrabipour et al., 2023). Therefore, we wanted to engineer chimeric proteins to test if these loops alone could mediate specificity switching. In our chimeras, we swapped both the RT (sequence: ^96^ESRTET^101^) and nSrc (^117^TEGD^120^) loops of Src with the RT (^73^VASGDN^78^) and nSrc (^95^HNGE^98^) loop sequences of Abl, and *vice versa* in each SH3 domain. We termed the resulting proteins, Src_Abl_ and Abl_Src_, where the subscript indicates the sequence identity of both the RT and nSrc loops. These proteins were also recombinantly expressed and purified as SUMO fusions and we determined binding affinities using fluorescence anisotropy experiments with our F*-PLLP and F*-p40 peptides, as described in the Materials and Methods (Figure 2; Table 1).

In our Src_Abl_ SH3 chimera, binding affinity for the Src-specific F*-PLLP peptide was reduced ∼14-fold, to *K*_D_ = 140 ± 20 μM, as compared to the wild-type Src SH3 domain (Figure 2A; Table 1). Interestingly, Src_Abl_ SH3 bound the Abl-specific F*-p40 peptide with similar affinity, *K*_D_ = 130 ± 40 μM, indicating that specificity was indeed altered by this substitution (Figure 2C; Table 1). In contrast, while binding to the Abl-specific F*-p40 peptide was reduced by > 60-fold for Abl_Src_ SH3, *K*_D_ = 210 ± 30 μM, this chimeric protein continued to show undetectable binding to the Src-specific F*-PLLP peptide, *K*_D_ > 1,000 μM (Figure 2D; Table 1).

### Structural analyses of SH3-ligand models

**FIGURE 3:**
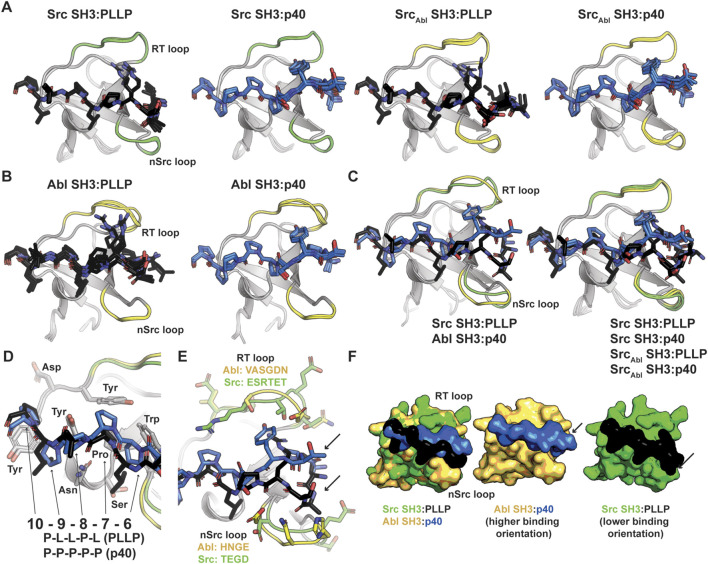
Alphafold–Multimer (ColabFold) models of Src, Abl, and SrcAbl SH3 domains with F*-PLLP and p40 ligands. **(A, B)** Alphafold–Multimer results for Src and SrcAbl **(A)** or Abl **(B)** SH3 domains with the PLLP (black sticks, colored by atom) or p40 (blue sticks, colored by atom) ligands. In all cases, the results for each complex align with RMSD values of <1.75 Å, with many even closer [e.g., Abl SH3:PLLP aligns with RMSD <1.3 Å). SH3 domains are shown in cartoon representation with the RT and nSrc loops colored based on the origin sequence, green for Src SH3 and yellow for Abl SH3. The PLLP and p40 peptides are shown in stick representation and colored by atom (C = black (PLLP) or blue (p40)]. **(C)** Although the Src and Abl RT and nSrc loops are in distinct conformations (left figure), alignment of the SrcAbl chimeric SH3 domain with the wild-type Src SH3 domain indicates that the loop conformations largely match the wild-type scaffold. Structures are rendered and colored as in **(A, B)**. **(D)** SH3 residues in Src and Abl that interact with the 6–10 ligand positions are identical. SH3 domains are in cartoon representation with side chain sticks for interacting residues (C = gray). Peptide positions are labeled. **(E)** The p40 peptide binds in a “higher” orientation and PLLP in a “lower” orientation. In the left figure, the SH3 domains are shown in cartoon, with side chain atoms of the RT and nSrc loops shown as sticks (C = green (Src) or yellow (Abl)). The peptides are rendered as in **(A–D)**. **(F)** Surface representation of **(E)**. Here, arrows indicate the differing peptide orientations for PLLP (black, with Src in green) and p40 (blue, with Abl in yellow). In all stick representation images, O = blue, N = red.

To better understand our biochemical assay results, we generated structural models of our SH3-ligand complexes using Alphafold–Multimer on the ColabFold server (Kaneko et al., 2008b; Donaldson et al., 2002). We created models of wild-type Src and Abl SH3 domains with each peptide, PLLP and p40, as well as our chimeric Src_Abl_ and Abl_Src_ SH3 proteins with each peptide (Figures 3A, B; Table 2). Because of relatively poor confidence measures, we were unable to analyze models of Abl_Src_ bound to either peptide (Table 2). Overall, the 5 output models generated for each complex were internally consistent, with alignments of <0.18 Å in all cases (Figures 3A, B). However, there were notable exceptions, which included steric clash based on the PyMOL library for several of the peptides with SH3 domains. Therefore, our analyses will be based on models without steric clash.

**TABLE 2:** Confidence measures from AlphaFold–Multimer predictions (for the rank_001 model plus a standard deviation for all 5 output models in parentheses). Italics indicate that these predictions are either not robust based on the confidence measures, or that only certain models (e.g., rank_001) can be interpreted; therefore, these models were excluded from analyses.

	pLDDT	pTM	ipTM
Src SH3:PLLP	94.3 (0.95)	0.838 (0.014)	0.775 (0.024)
Src SH3:p40	91.8 (1.08)	0.801 (0.009)	0.749 (0.018)
Src_Abl_:PLLP	92.7 (0.92)	0.818 (0.014)	0.728 (0.028)
Src_Abl_:p40	91.8 (1.08)	0.801 (0.009)	0.749 (0.018)
W122C Src SH3:PLLP	90.6 (1.59)	0.782 (0.020)	0.715 (0.049)
W122C Src SH3:p40	89.1 (1.59)	0.754 (0.012)	0.697 (0.034)
W122C Src_Abl_ SH3:p40	88.3 (1.14)	0.752 (0.008)	0.706 (0.032)
Abl SH3:PLLP	91.8 (1.72)	0.806 (0.017)	0.669 (0.049)
Abl SH3:p40	94.9 (1.15)	0.841 (0.013)	0.847 (0.018)
Abl_Src_ SH3:PLLP	89.4 (1.81)	0.779 (0.016)	*0.523 (0.108)*
Abl_Src_ SH3:p40	95.5 (2.26)	0.839 (0.023)	*0.839 (0.126)*
C100W Abl SH3:p40	96.0 (0.93)	0.853 (0.011)	0.859 (0.016)

We observed RT and nSrc loop differences between the Abl and Src SH3 domains, however, our Src_Abl_ chimeric protein loops are consistent with the Src parent/wild-type protein (Figure 3C). The PXXP motifs at the C-terminus of each peptide (PLLP and PPPP, respectively, and referred to as positions P7-P10, as described above and based on amino acid identity) interact largely with the βA-βB loop and side-chains in the 3_10_ helix in a consistent manner between the two peptide sequences and other SH3 domains (Pisabarro et al., 1998). All PxxP-interacting residues are conserved between Abl and Src SH3 (Figure 3D). At the N-terminal end of the peptide, we observed differing peptide conformations for the first 6 amino acids for each peptide sequence (Figures 3E, F), with an upward translation of p40 binding as compared to PLLP, including for the Src_Abl_ chimera (Figure 3C).

**FIGURE 4:**
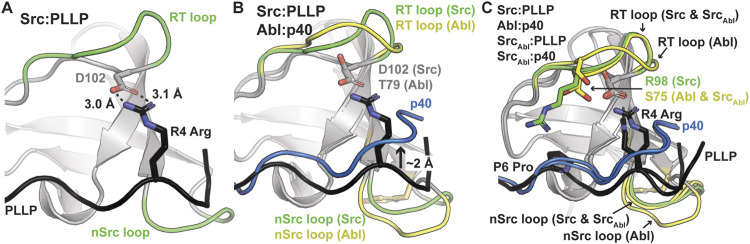
Ligand interactions with the RT and nSrc loops of Src and Abl SH3 domains. In all, SH3 domains and ligands are shown in cartoon representation, with loops colored by original sequence (green for Src and yellow for Abl), even in the chimeric proteins, as labeled. The ligands are shown as black for PLLP and blue for p40. Relevant side chains are shown as sticks and colored by atom (C = as above, N = blue, O = red). **(A)** The P4 Arg in the ligand interacts with D102 in Src SH3. Distances shown as black dashed lines and labeled. **(B)** The p40 ligand bound to Abl SH3 is translated ∼2 Å in the ligand binding pocket. **(C)** Although the RT loop of Src_Abl_ SH3 is in a similar conformation as wild-type Src SH3, R98 (Src) is replaced by S75 (Abl), allowing the p40 peptide to bind Src_Abl_ in the elevated peptide-binding conformation. The nSrc loop of Src_Abl_ is also in a Src-like conformation.

As others have previously identified as well, these translation differences in peptide binding are likely due to an electrostatic interaction between the R4 Arg of the PLLP sequence and D102 Src SH3, using full-length Src numbering (Figure 4A). This interaction is a well-studied characteristic of Src SH3 specificity (Saksela and Permi, 2012; Shah et al., 2018; Musacchio et al., 1994), and appears to result in the lowered translation of the peptide in the binding cleft (Figure 4B). The D102 amino acid immediately follows the RT loop. In Abl, this position corresponds to T79. Because of the R4 residue, an upward translation of PLLP in the Src SH3 binding cleft would result in steric clash with the RT loop, as the main chain atoms of the peptide are translated ∼2 Å between the two peptide conformations (Figure 4B). Our modeling results also revealed that the RT and nSrc loops of Src_Abl_ are in a Src-like conformation, despite containing sequences from Abl SH3 (Figure 4C).

### The WX sequence motif at the base of the nSrc loop influences nSrc loop orientation

**FIGURE 5:**
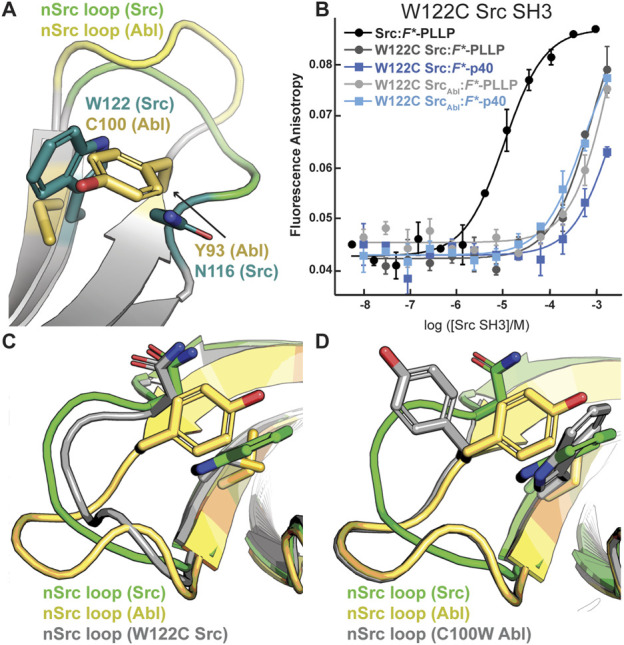
The WX motif following the nSrc loop determines its conformation. All structures, generated by Alphafold–Multimer, are shown in cartoon representation and colored as labeled. Side chain sticks are shown for relevant positions, and colored by atom (C = yellow (Abl), green (Src), or gray (W122C Src or C100W Abl), O = red, N = blue). **(A)** The X position of the WX motif in Src (X = W) and Abl (X = C) makes intra-SH3 interactions with N116 (Src) or Y93 (Abl). **(B)** Binding affinity data by fluorescence anisotropy experiments in triplicate confirms an importance for the W122 residue in the Src SH3 domain (Table 1). All averaged curves were normalized to an identical starting value to better illustrate K_d_ shifts. **(C, D)** The nSrc loop in the W122C Src model is shifted with respect to wild-type Src SH3 **(C)**; whereas the nSrc loop in the C100W Abl SH3 model maintains an “Abl-like” conformation **(D)**.

Although the peptides bind in differing orientations with respect to the peptide-binding cleft, we wanted to further investigate the relative conformation of the RT and nSrc loops in Src versus Src_Abl_, which include the sequences derived from Src or Abl, respectively (Figure 4C). Structural analyses of our Alphafold-generated models suggested that an intra-SH3 interaction on either side of the nSrc loop may modulate its loop conformation, independent of the sequence identity of the loop itself. Specifically, an aromatic Trp (W122) C-terminal to the nSrc loop is counterbalanced by an aromatic Tyr (Y93) N-terminal to the nSrc loop in Abl SH3 (Figure 5A). Because these positions are outside the nSrc loop, we reasoned this may be why the Abl sequence in Src_Abl_ maintains a Src-like nSrc loop conformation in our models (Figure 4C). As we do not see specific W122 interactions with the side chain atoms of N116, we predicted that the W122 in Src SH3 (analogous position is C100 in Abl SH3) may be primarily responsible for this observation.

To test this hypothesis, we recombinantly expressed and purified a W122C mutation in Src SH3 and Src_Abl_ SH3, as described in the Materials and Methods. While W122C Src SH3 showed little to no binding for either peptide, the W122C Src_Abl_ SH3 domain was able to bind F*-p40 weakly, *K*_D_ = 450 ± 190 μM (Figure 5B; Table 1). We also recombinantly expressed and purified C100W Abl SH3 and C100W Abl_Src_; however, the C100W Abl_Src_ SH3 protein was unstable in our purification. We saw that C100W Abl SH3 revealed undetectable binding to F*-PLLP, defined as >1,000 μM in our assay. Binding of C100W Abl SH3 to F*-p40 was weaker by ∼18-fold as compared to the wild-type protein, *K*_D_ = 55 ± 20 μM, but was still relatively high for the SH3 domain interactions tested (Figure 5B; Table 1).

Protein structure prediction models generated using Alphafold–Multimer show differences for nSrc loop conformation in the W122C Src SH3 and C100W Abl SH3 mutations. Based on our models, the W122C mutation in Src SH3 allows the nSrc loop to adopt a more “Abl-like” conformation, although it is still mostly “Src-like” (Figure 5C). In contrast, we see no difference in the nSrc loop for a C100W Abl SH3 model as compared to the wild-type protein (Figure 5D). This is consistent with our binding affinity results.

### Molecular dynamics simulations investigate the relative flexibility in the nSrc loop upon mutation of the WX motif

**FIGURE 6:**
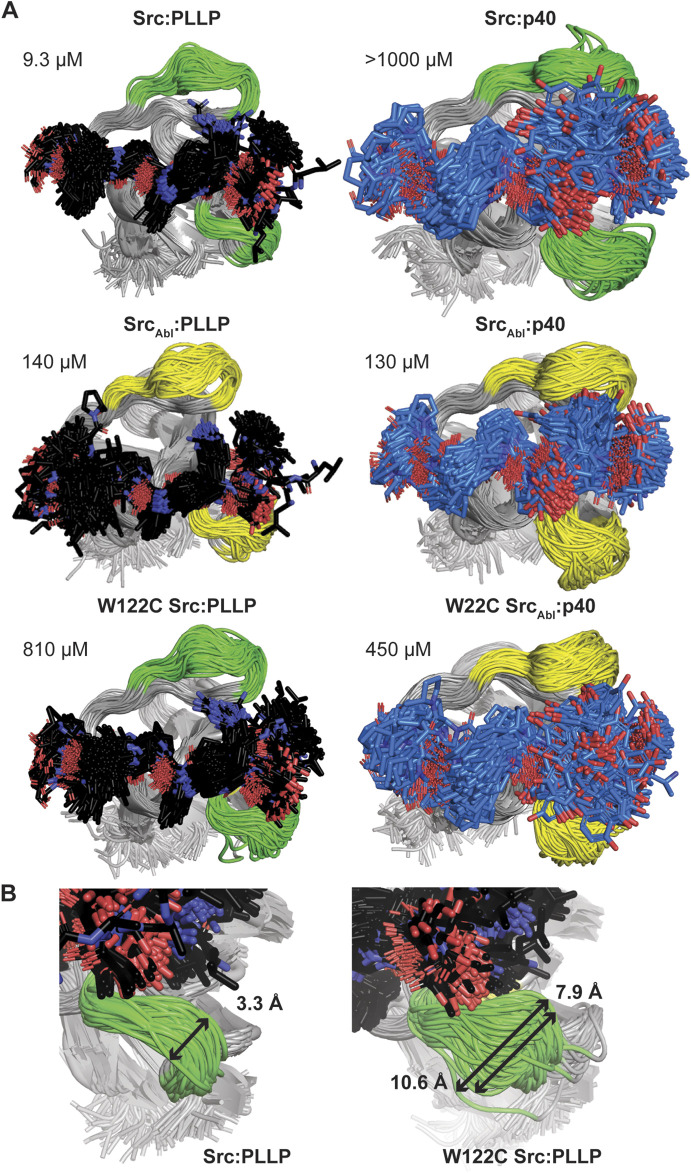
Molecular dynamics simulations of Src SH3-peptide complexes. All MD simulations were run for 1 μs. **(A)** For each simulation, 200 states (Δt = 5 ns) are shown aligned by SH3 domain, which are shown in cartoon representation and labeled. The RT and nSrc loops are colored by sequence identity (green = amino acids from Src, yellow = amino acids from Abl). The bound peptides are shown in stick representation and colored by atom (C = black for PLLP, marine for p40, O = red, N = blue). Calculated binding affinities (Table 1) are displayed. **(B)** The relative positions sampled by the nSrc loop in the Src:PLLP (left) and W122C Src:PLLP (right) simulations are shown, with distances measured (indicated by a black arrow and as labeled). Structures are rendered as in **(A)**.

Because we were unable to generate an Abl SH3 variant that showed binding to the Src target, PLLP, and based on our C100W Abl SH3 results, we decided to focus on nSrc loop conformational differences in Src SH3 for additional study. To investigate the relative stability of our AlphaFold models and further investigate the WX motif, we ran 1 μs molecular dynamics (MD) simulations of several of our Src SH3-peptide complexes, including (with relative binding affinities, as reported in Table 1, in parentheses): Src-PLLP (9.3 μM), Src-p40 (>1,000 μM), Src_Abl_-PLLP (140 μM), Src_Abl_-p40 (130 μM), W122C Src-PLLP (810 μM), and W122C Src_Abl_-p40 (450 μM) (Figure 6A; Supplementary Figure S1).

The Src SH3 domain backbone remained relatively stable during the length of all simulations, as assessed by calculated root mean square deviation (RMSD) values, compared to the initial structure (Supplementary Figure S1A). Alignment of 200 states from each simulation, including a state every 5 ns, revealed that in general, the PLLP peptide is more stable in the pocket than p40 (Figure 6A). Because all but one of these interactions are of relatively low affinity (>100 μM), it is challenging to use these MD simulations (or the AlphaFold models) to make predictions about why one peptide will bind over another. For example, we included the Src SH3:p40 complex as a control (affinity >1,000 μM or undetectable in our assay) (Table 1); however, our MD simulation does show binding, although the 1-6 positions of the peptide are relatively unstable in the pocket (Figure 6A). This variability is also observed in the W122C Src_Abl_-p40 simulation (Figure 6A). These factors aside, we do conclude that PLLP is more stable in the binding pocket because of the R4 Arg-D102 interaction previously described (Figure 4A).

Ultimately, our MD simulations do allow us to assess relative flexibility in the nSrc loop upon mutation. Notably, we see the largest root mean square fluctuations (RMSF), as compared to the average, in Cα atoms for specific RT and nSrc loop residues in all simulations (Supplementary Figure S1B). In the RT loop, the largest RMSF values were seen for R98 in the Src-PLLP and W122C Src-PLLP simulations (Supplementary Figure S1B). In the nSrc loop, the largest RMSF values were seen for E118 (or E118N in Src_Abl_) in Src_Abl_-PLLP, Src_Abl_-p40, W122C Src_Abl_-p40, and W122C Src-PLLP. Comparing Src-PLLP and W122C Src-PLLP states throughout our simulations confirms this result (Figure 6B). Measurements between the loops farthest from each other in our Src-PLLP simulation revealed a distance of 3.3 Å. For W122C Src-PLLP, this distance was 10.6 Å (7.9 Å without an outlier loop) (Figure 6B). Taken together, this suggests that the single W122C mutation may increase flexibility in the nSrc loop of the Src SH3 domain.

### Investigating a loop orientation-determining residue in the WX sequence motif

**FIGURE 7:**
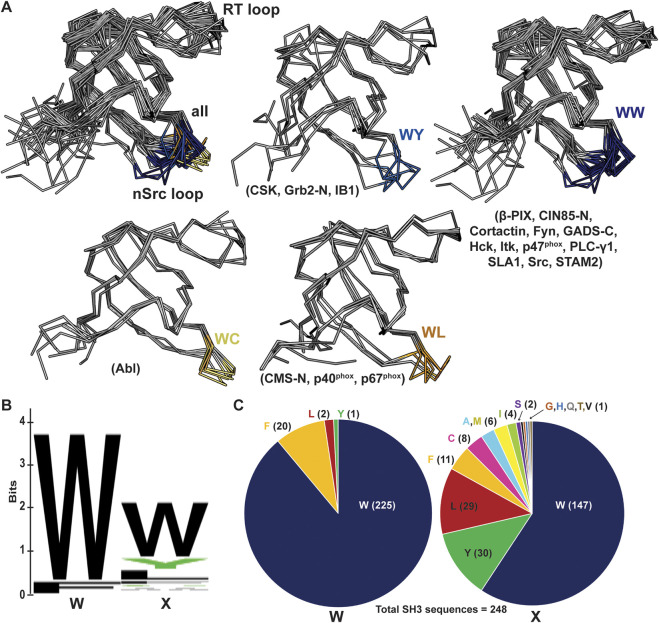
nSrc loop conformations and sequences of multiple SH3 domain structures. **(A)** All structures are shown in ribbon representation, with the nSrc loop residues colored based on the “WX” motif immediately following the loop (WW = dark blue, WY = blue, WC = yellow, WL = orange). The SH3 domains that are represented are indicated in parentheses; the majority of these structures include ligand, most in the Class II orientation. PDB ID codes used include: 1BBZ, 1JU5, 1AB0, 1OPL, 2FO0, 1OPK, 2J6F, 2J6O, 1K4U, 1W70, 1AWJ, 1YWO, 1RLP, 1H3H, 1UTI, 1UJ0, 1ZSG, 2P4R, 1CKA, 2DF6, 1AVZ, 1EFN, 1OEB, 2D0N, 2JT4, 2D1X, 2AK5, 2BZ8, 1WLP, 2HCK, 1AD5, 1FMK, 1Y57, 2SRC, 1KSW, 1JEG, 1AZE, 4GBQ, 2FPD (10, 16, 18, 19, 24–54). **(B, C)** The same data shown as a **(B)** WebLogo of the WX motifs for 253 SH3 domain sequences from UniProt, and **(C)** pie charts of the sequence composition per position. Of the 248 sequences analyzed, 91% contain a Trp in the first position and 59% in the second position.

We next wanted to use existing SH3 structures to assess if this position may broadly affect loop orientation. We analyzed 39 previously solved SH3 structures, most of which had peptides bound in either the Class I (4 structures) or Class II (35 structures) orientation (Kaneko et al., 2008b). We aligned these structures and colored the nSrc loop residues based on the WX motif immediately following the nSrc loop, including blue(s) for X = aromatic and yellow/orange for X = Cys or Leu (Figure 7A). Here, the X residues are the W122 Src SH3 and C100 Abl SH3 positions previously discussed. Although there are multiple structures for several of the SH3 domains, we have representative structures included for SH3 domains from the following proteins: WC (Abl), WL (CMS N-terminal SH3, or CMS-N, p40^phox^, p67^phox^), WY (CSK, Grb2-N, IB1), and WW (β-PIX, CIN85-N, Cortactin, Fyn, GADS C-terminal SH3 or GADS-C, Hck, Itk, p47^phox^, PLC-γ1, SLA1, Src, STAM2) [(Pisabarro and Serrano, 1996; Pisabarro et al., 1998; Pisabarro and Serrano, 1996; Pisabarro et al., 1998; Nagar et al., 2003; Pisabarro et al., 1998; Donaldson et al., 2002; Musacchio et al., 1994; Nagar et al., 2006; Moncalián et al., 2006; Jozic et al., 2005; Kami et al., 2002; Massenet et al., 2005; Andreotti et al., 1997; Deng et al., 2005; Liu et al., 2003; Lewitzky et al., 2004; Kaneko et al., 2003; Mott et al., 2005; Janz et al., 2007; Wu et al., 1995; Hoelz et al., 2006; Arold et al., 1997; Lee et al., 1996; Wittekind et al., 1997; Harkiolaki et al., 2003; Dimasi, 2007; He et al., 2007; Hashimoto et al., 2006; Ogura et al., 2006; Xu et al., 1997; Cowan-Jacob et al., 2005; Witucki et al., 2002; Ghose et al., 2001; Vidal et al., 1999; Kristensen et al., 2006; Xu et al., 1999)].

Our analyses revealed that SH3 domains with WW or WY motifs following the nSrc loop contain more “Src-like” conformations whereas those with WC or WL motifs appear more “Abl-like.” Of the structures analyzed, the WY motif-containing SH3 domains show the most variability (Figure 7A). Notably, these results are based on static structures available in the Protein Data Bank (PDB). It would be interesting to run MD simulations or conduct NMR experiments on a variety of WX motif-containing SH3 domains to test the influence of the WX loop on nSrc loop flexibility, as observed in our MD simulations with Src SH3 versus W122C Src SH3 (Figure 6B). Overall, our results suggest that this position may play an important role in the conformation (and potentially the relative flexibility) of the nSrc loop. In combination with RT loop residues, this directly determines how ligands could interact with the peptide-binding cleft and previously identified specificity pockets for SH3 domains.

Finally, we analyzed the sequences of all 344 annotated SH3 domains in the human proteome, using the UniProt database [(Magrane and Consortium, 2011; UniProt Consortium, 2021; UniProt Consortium, 2019)]. We extracted the SH3 domain sequences, performed a multiple sequence alignment using Clustal Omega, and visualized the results using Jalview (Supplementary Figure S2) [(Sievers et al., 2011; Clamp et al., 2004)]. There were several atypical SH3 domains based on secondary structural elements according to manual visualization of available AlphaFold structures [(Varadi et al., 2022; Jumper et al., 2021)]; therefore, we chose to exclude any sequences that did not align properly from further analyses. In total, we analyzed the WX sequence motifs for 248 human SH3 domains (Figures 7B, C), and found that indeed, a WW sequence is the most observed, with 138 occurrences (55.6% of total sequences). Position by position, we see a Trp in the first position of the sequence motif in 91% of SH3 domains analyzed, and 59% in the second position (Figure 7C). While there were four alternative residues in the first position (including Leu, Phe, and Tyr), there are an additional thirteen alternatives in the second position (Ala, Cys, Phe, Gly, His, Ile, Leu, Met, Gln, Ser, Thr, Val, Tyr) (Figure 7C). Overall, this result confirms variability in the X, or second, position of the WX sequence motif defined here, within the human SH3 domains.

## Discussion

Despite their relatively small size, SH3 domains display remarkable plasticity in ligand binding and the family has been described as *versatile* and *diverse* [(Mehrabipour et al., 2023; Saksela and Permi, 2012; Kaneko et al., 2008b; Kaneko et al., 2011)]. This includes the ability to bind ligands in opposite orientations, depending on the sequences of the short linear motifs (SLiMs) recognized. Specificity-determining components of SH3 have been described, and the RT and nSrc loops are known to play an important role [(Saksela and Permi, 2012; Kaneko et al., 2008b; Kaneko et al., 2011)]. A conserved Trp residue immediately following the nSrc loop, and at the start of the βC strand was also identified as forming a hydrophobic pocket that is critical for ligand binding (Kaneko et al., 2011). Here, we extend discussion of this Trp residue to include its C-terminal neighbor, and define the WX motif, where the identity of the X residue is also important for modulating the conformation (and potentially flexibility) of the nSrc loop.

Our biochemical and computational analyses suggest that the WX motif may determine the conformation of the nSrc loop of Src SH3, even in the presence of peptides that bind in distinct conformations (e.g., PLLP versus p40). When we broadened this analysis to include 39 SH3 domain structures, we also saw clear patterns in the positions of the nSrc loops (Figure 7), supporting our biochemical and modeling data. Multiple sequence analyses of the WX sequence motifs of 253 human SH3 domains confirmed that the X position is variable, with a total of fourteen amino acids in these sequences.

While this study was relatively limited in scope, our work identifies an important residue for loop positioning (and potential flexibility), and therefore SH3 specificity. It would be interesting to further test these observations using additional SH3 domains and a variety of ligand sequences. Future work could also include broad mutational characterization and analyses of the X position in the WX sequence motif identified, as well as high throughput studies of the specificity profiles of these differing SH3 domains. Critically, experimental analyses of the effect of the WX motif on relative flexibility of the nSrc loop is needed to confirm our computational data. Considering their widespread and critical roles in the cell, a better understanding of the molecular determinants of SH3 specificity can advance knowledge of complex signaling pathways towards the improvement of human health.

## Materials and methods

### Protein expression and purification

Sequences corresponding to the Abl and Src SH3 (residues 84-145, UniProt ID SRC_HUMAN) domains were used for the wild-type SH3 domains. All proteins were expressed as His_6_-SUMO fusion proteins using the pET28a (+) plasmid (Genscript). Expression and purification protocols were similar to those used previously for other SLiM-binding domains [(Tahti et al., 2023; Gao et al., 2020; Wofford et al., 2021)]. All sequences used are in the **Supporting Information**.

Briefly, plasmids were transformed into BL21 DE3 chemically competent *Escherichia coli* cells. Selected colonies were grown in Terrific Broth (TB) at 37°C with shaking at 210 rpm. Once an optical density (OD) of 0.6–0.8 at λ = 600 nm was reached, protein expression was induced by the addition of 0.15 mM isopropyl-β-D-1-thiogalactopyranoside (IPTG) for 16–18 h at 18°C. Cells were harvested via centrifugation at 3,000x*g* for 10 min, followed by resuspension in lysis buffer (0.05 M Tris pH 7.5, 0.2 M NaCl, 0.01 M MgCl_2_, 0.01 M CaCl_2_, 0.05 M imidazole pH 7.5, 20% (*w/v*) glycerol, 0.25 mM TCEP; 20 μg/mL DNAse and Complete EDTA-free protease inhibitor tablets (1 tablet/50 mL lysis buffer) were also added). Cells were lysed using sonication at 4°C and whole cell lysate was clarified with centrifugation at 17,500 rpm at 4°C for 30 min. The filtered supernatant was applied to a NiNTA HisTrap 5 mL column (Cytiva) with wash buffer [0.025 M Tris pH 7.5, 0.25 M NaCl, 10% (*w/v*) glycerol, 0.025 M imidazole pH 7.5, 0.25 mM tris(2-carboxyethyl)phosphine (TCEP)]. Protein was eluted into fractions using elution buffer [0.025 M Tris-HCl pH 7.5, 0.05 M NaCl, 10% (*w/v*) glycerol, 0.4 M imidazole pH 7.5, 0.25mM TCEP]. Pooled fractions were dialyzed in the presence of ULP-1 SUMO protease in dialysis buffer [0.025 M Tris pH 7.5, 0.15 M NaCl, 10% (*w/v*) glycerol, 0.5mM TCEP] overnight, and then run over another NiNTA His-Trap 5 mL column to separate the SH3 domain from the His_6_-SUMO tag, using running buffer plus 0.025 M imidazole pH 7.5. The flow-through was concentrated using Amicon Ultra 3K Centrifugal Filters and further purified using a Superdex S75 16/600 column, with running buffer. SDS-PAGE was used to assess protein purity and the absorbance at λ = 280 nm with the calculated extinction coefficient (s), by Expasy ProtParam, was used to calculate protein concentration (Wilkins et al., 1999).

Calculated extinction coefficients used were as follows: SUMO-Src-SH3 = 18,450 M^−1^ cm^−1^ (cleaved, 16,960 M^−1^ cm^−1^); SUMO-Abl-SH3 = 16,960 M^−1^ cm^−1^ (cleaved, 15,470 M^−1^ cm^−1^); SUMO-Src_Abl_-SH3 = 18,450 M^−1^ cm^−1^ (cleaved, 16,960 M^−1^ cm^−1^); SUMO-Abl_Src_-SH3 = 16,960 M^−1^ cm^−1^ (cleaved, 15,470 M^−1^ cm^−1^); W122C SUMO-Src-SH3 = 12,950 M^−1^ cm^−1^ (cleaved, 16,460 M^−1^ cm^−1^); C100W SUMO-Abl-SH3 = 22,460 M^−1^ cm^−1^ (cleaved, 20,970 M^−1^ cm^−1^); W122C SUMO-Src_Abl_-SH3 = 12,950 M^−1^ cm^−1^ (cleaved, 11,460 M^−1^ cm^−1^).

### Fluorescence anisotropy assays

*The* peptides used for fluorescence anisotropy assays were F*-PLLP (sequence: FITC-Ahx-LASRPLPLLP), the Src SH3-specific binder, and F*-p40 (FITC-Ahx-APTYSPPPPP), the Abl SH3-specific binder (Biomatik) (*6*, *20*). Fluorescence anisotropy assays were performed using a BioTek Synergy H1 plate reader, as previously described [(Tahti et al., 2023; Gao et al., 2020; Wofford et al., 2021; Valgardson et al., 2019)]. Solution conditions included running buffer plus 0.1 mg/mL bovine serine albumin (BSA) and 0.5 mM Thesit, with 30 nM fluorescent peptide. Determined *K*_D_ values were the average calculated from triplicate experiments (Cushing et al., 2008). *K*_d_ values were determined as previously, and as the midpoint between the anisotropy resulting from the free reporter peptide and SH3-peptide complex [(Cushing et al., 2008; Vouilleme et al., 2010; Amacher et al., 2013; Amacher et al., 2014)].

### Molecular dynamics simulations

A chosen AlphaFold3 output model (usually, the ranked_001 model, but an additional model if steric clash was observed) was capped with an N-terminal acetyl and C-terminal N-methyl group, respectively. MD simulations were performed using GROMACS 2022.4 (Bauer et al., 2022). Each system was centered within a simulation box, maintaining a solute-box distance of 1.0 nm, and solvated using the TIP3P water model. The system was then given the solvent configuration spc216, representing a pre-equilibrated configuration of 216 water molecules based on the Simple Point Charge (SPC) (Berendsen et al., 1981). To mimic physiological conditions, ions were added to achieve a final concentration of 0.15 M, with charge neutrality maintained by a balanced addition of sodium and chloride ions. Simulations employed the AMBER99SB-ILDN force field (Wang et al., 2004). Energy minimization was performed using the steepest descent algorithm, with convergence defined by a maximum force of <1,000 kJ/mol/nm. Long-range electrostatic interactions were calculated using the particle mesh Ewald (PME) algorithm (Essmann et al., 1995), while short range electrostatic and Van der Waals cutoffs were set at 1.0 nm. A preliminary equilibration was conducted over 100 ps using an NVT file with parameters set for output control, electrostatics, bond parameters, neighbor searching, and temperature coupling. Pressure coupling was disabled. Temperature coupling employed a modified Berendsen thermostat, maintaining a reference temperature of 300 K across two coupling groups. The Verlet cutoff scheme was used for neighbor searching with a grid-cell based method. An additional equilibration phase was preformed using the isotropic Parrinello-Rahman barostat, targeting a reference temperature of 1 bar (Parrinello and Rahman, 1981). Production runs were conducted for 5 ns, with a timestep (dt) of 2fs. Each production run was then extended by an additional 995 ns. Final simulations were then analyzed and visualized in PyMOL (Schrödinger software).

### Programs used for structural modeling and analyses

AlphaFold–Multimer (in the CoLabFold notebook) was used to model ligand-bound SH3 domains, using the default settings [(Evans et al., 2021; Mirdita et al., 2022)]. Confidence measures are included in Table 2, for reference, pLDDT is a confidence score (>90 = modeled with high accuracy), pTM is an integrated measure (>0.5 = overall predicted fold for complex is similar to true structure), and ipTM correlates to the accuracy of predicted relative positions between multimers (>0.8 = confident high-quality predictions, 0.6-0.8 = “grey zone,” <0.6 likely represent a failed prediction). Binding curves were visualized using KaleidaGraph (Synergy software). Structural figures were rendered using PyMOL. We used UniProt to download the human SH3ome, following by Clustal Omega to generate a multiple sequence alignment (default settings), Jalview to view the results, and WebLogo and Excel to analyze and visualize the data (Supplementary Figure S2) [(Magrane and Consortium, 2011; UniProt Consortium, 2021; UniProt Consortium, 2019; Sievers et al., 2011; Clamp et al., 2004; Crooks et al., 2004)]. In our initial curation of the human SH3 multiple sequence alignment, we used the AlphaFold protein database to manually visualize WX sequence motifs that did not properly align [(Varadi et al., 2022; Jumper et al., 2021)].

## Data Availability

The raw data supporting the conclusions of this article will be made available by the authors, without undue reservation.
