# Supplementary material for: The conformation of the nSrc specificity-determining loop in the Src SH3 domain is modulated by a WX conserved sequence motif found in SH3 domains
Source: Front Mol Biosci. 2024 Dec 3;11:1487276. doi: 10.3389/fmolb.2024.1487276 (PMC11653366; doi:10.3389/fmolb.2024.1487276)
Supplement: Supplementary file 1 [file DataSheet1.pdf]

Supporting Information for:

The conformation of the nSrc specificity-determining loop in the Src SH3 domain is modulated by a WX sequence motif found in SH3 domains

Frederick Longshore-Neate, Caroline Ceravolo, Cole Masuga, Elise F. Tahti, Jadon M. Blount, Sarah N. Smith, Jeanine F. Amacher

Table of Contents:

Figure S1. Molecular dynamics simulations of Src SH3 and variant complexes. 2

Figure S2. Alignment of human SH3 domains. 3

Protein sequences used. 7

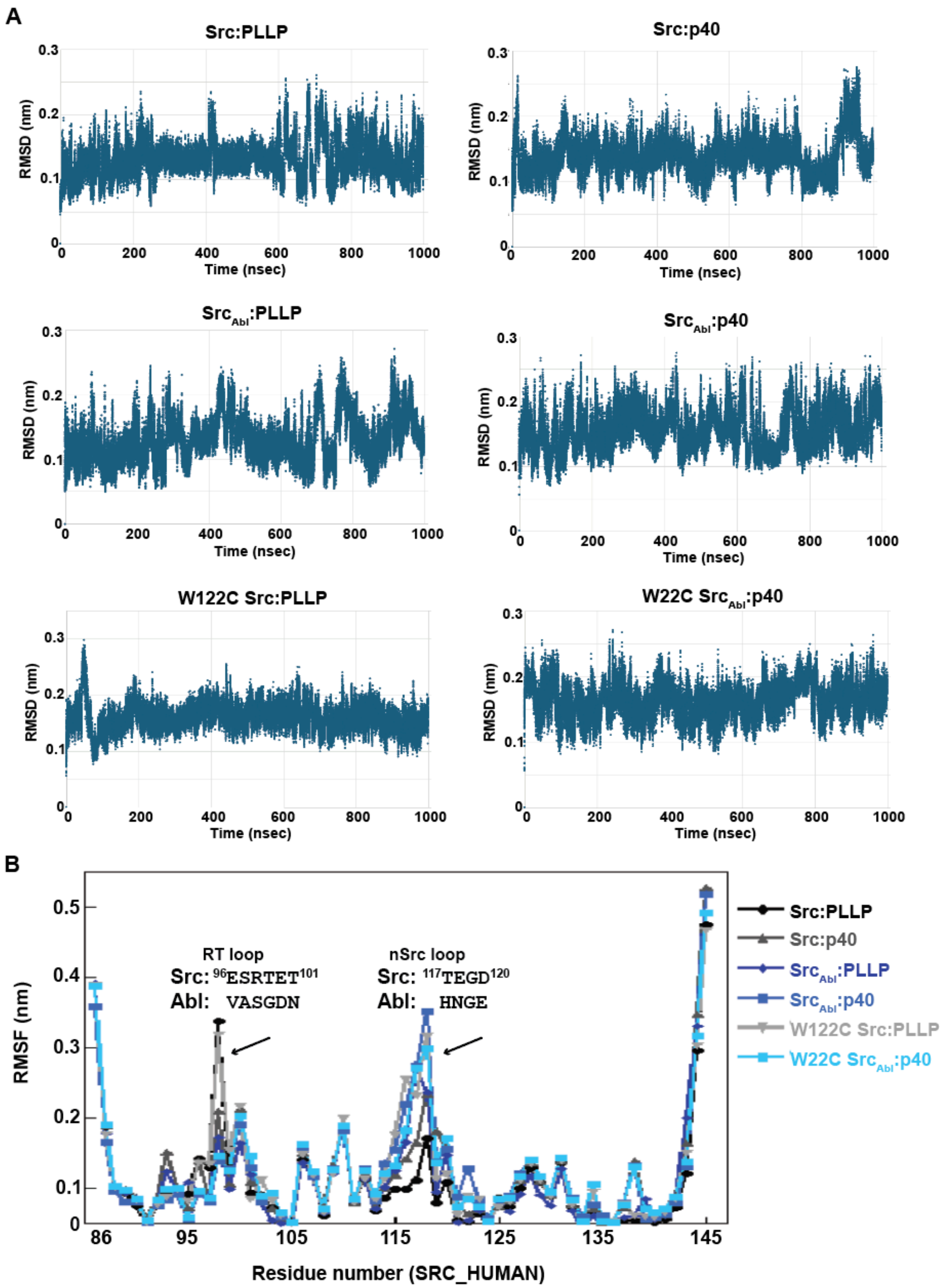

Figure S1. Molecular dynamics simulations of Src SH3 and variant complexes. (**A**) Root mean square deviation (RMSD) values, as compared to the initial model, for backbone atoms of the Src SH3 and variants for all simulations run, as labeled. (**B**) Root mean square fluctuation (RMSF) values per amino acid (using wild-type Src numbering, based on UniProt ID SRC_HUMAN). Each simulation is colored as labeled, with Src variants that contain RT and nSrc loop sequences from Src in black/gray colors, and those with loop sequences from Abl in blue colors.

Figure S2. Alignment of 248 human SH3 domains extracted from the UniProt database. Where sequences were variable, e.g., differing lengths of the RT and nSrc loops, the alignment was truncated for clarify. Asterisks (*) are used to show where the alignment was edited. Asterisks (*) on the left also highlight the Abl (UniProt ID P00519) and Src (UniProt ID P12931) sequences.

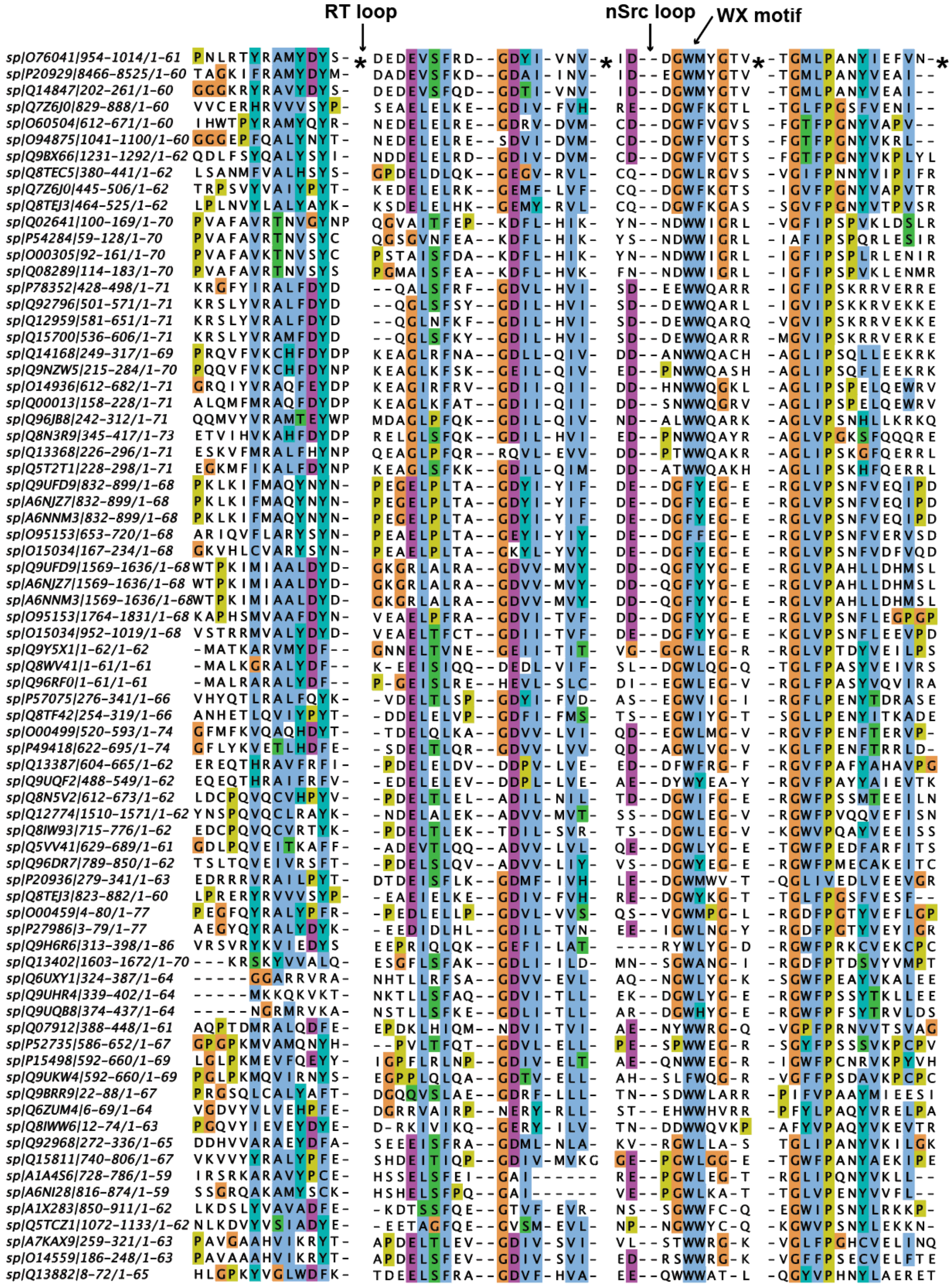

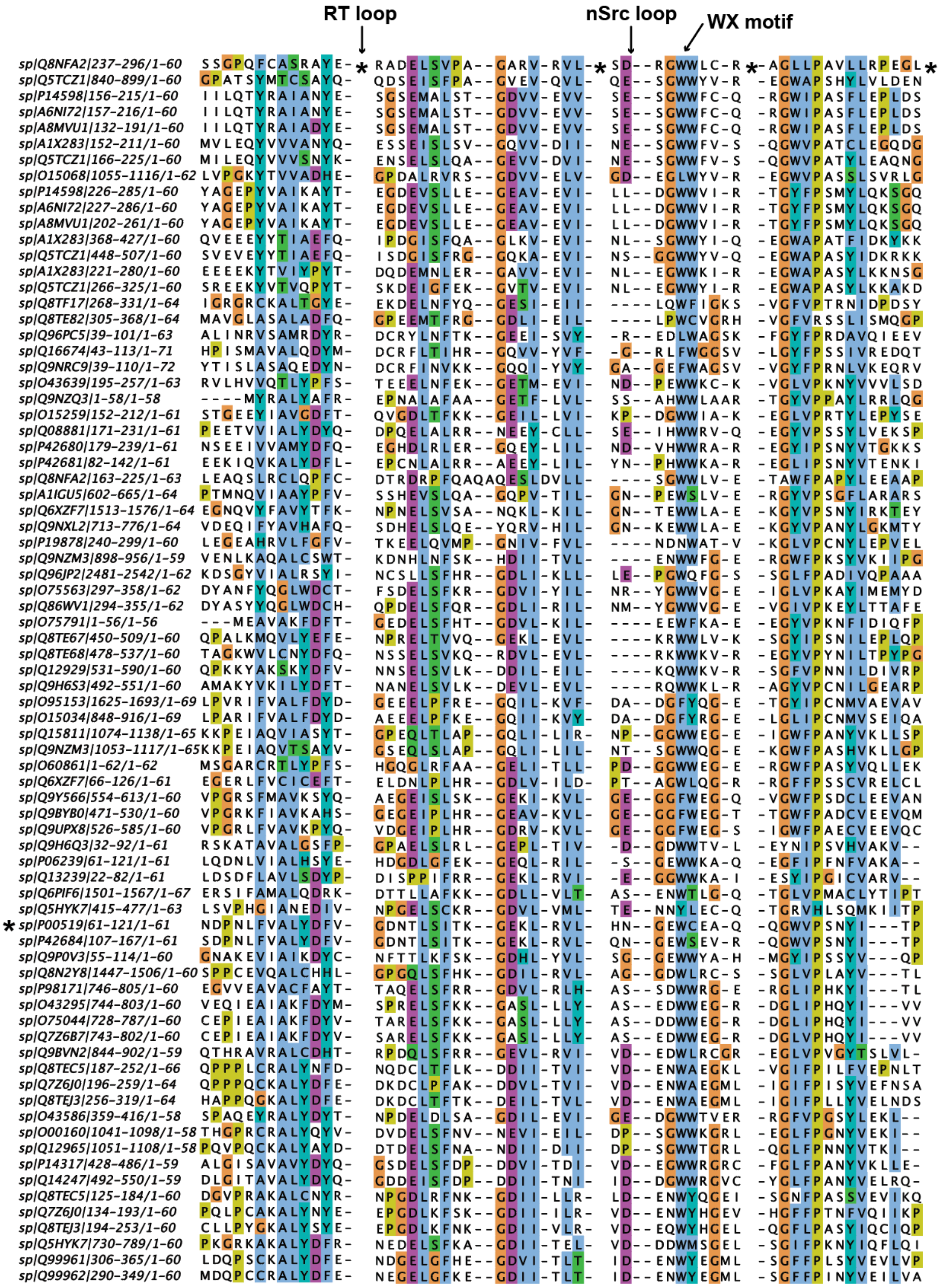
RT loop
nSrc loop
WX motif

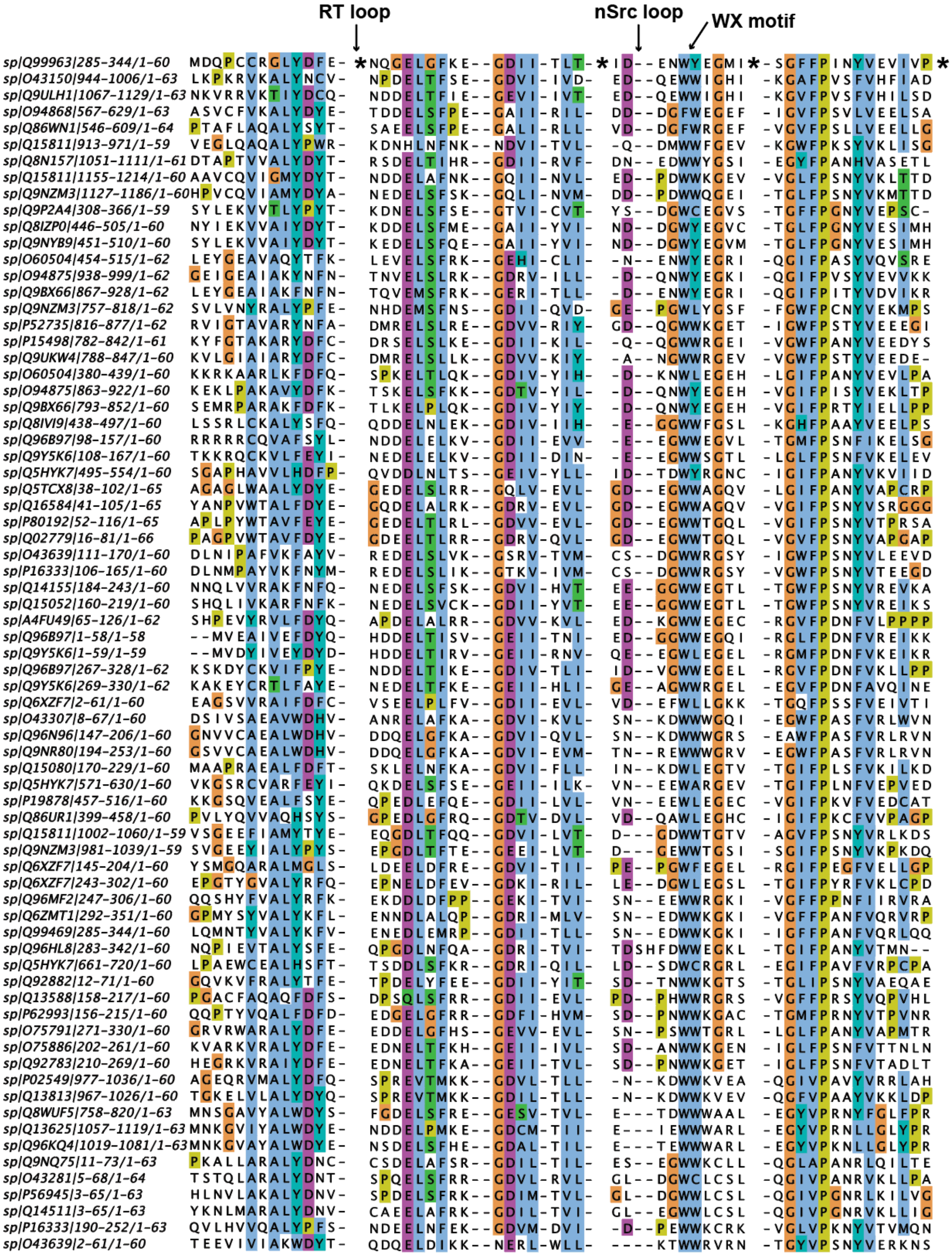
RT loop
nSrc loop
WX motif

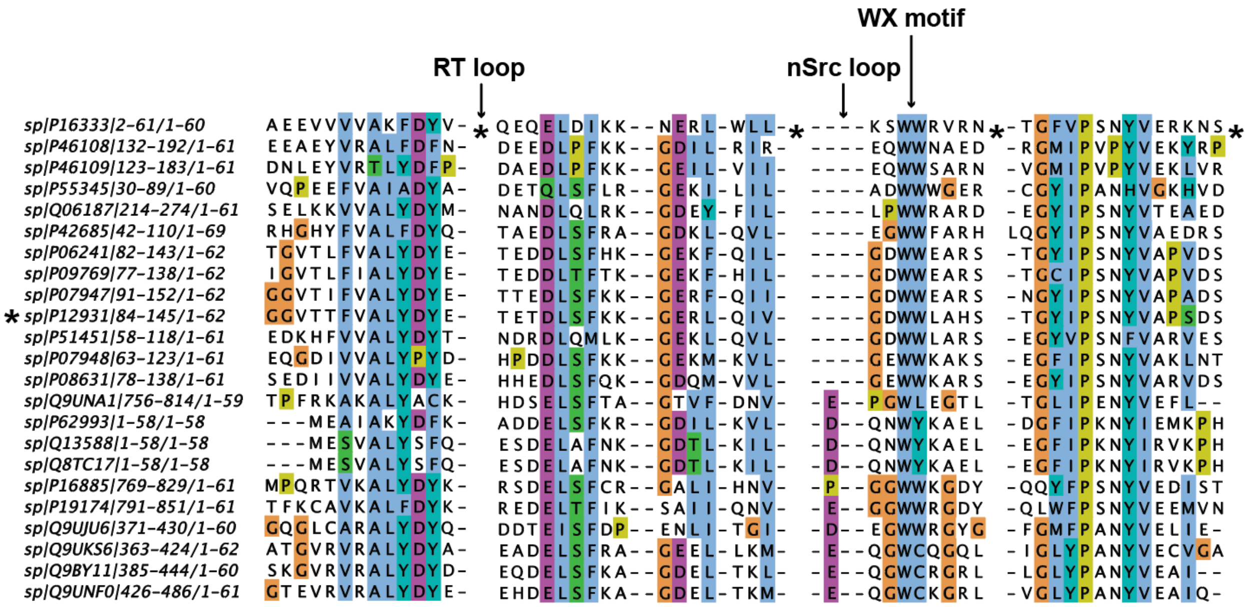
WX motif
RT loop
nSrc loop
sp|P16333|2-61/1-60
sp|P46108|132-192/1-61
sp|P46109|123-183/1-61
sp|P55345|30-89/1-60
sp|Q06187|214-274/1-61
sp|P42685|42-110/1-69
sp|P06241|82-143/1-62
sp|P09769|77-138/1-62
sp|P07947|91-152/1-62
sp|P12931|84-145/1-62
sp|P51451|58-118/1-61
sp|P07948|63-123/1-61
sp|P08631|78-138/1-61
sp|Q9UNA1|756-814/1-59
sp|P62993|1-58/1-58
sp|Q13588|1-58/1-58
sp|Q8TC17|1-58/1-58
sp|P16885|769-829/1-61
sp|P19174|791-851/1-61
sp|Q9UJU6|371-430/1-60
sp|Q9UKS6|363-424/1-62
sp|Q9BY11|385-444/1-60
sp|Q9UNF0|426-486/1-61

Recombinant protein sequences used in this study. The pET28a(+) plasmid was used for all. The 6xHis-tag and TEV protease site included are in **bold** and the SUMO (*Saccharomyces cerevisiae* Smt3, NCBI reference: NP_010798.1) sequence is underlined. In chimeric constructs and mutants, the changed residue(s) is/are in **red**.

>SUMO_Src_SH3
MGSSHHHHHHGSGLVPRGSASMSDSEVNQEAKPEVKPEVKPETHINLKVSDGSSEIFFKIKKTTPLRRLM
EAFAKRQGKEMDSLRFLYDGIRIQADQTPEDLDMEDNDIIEAHREQIGGVTTFVALYDYESRTETDLSFK
KGERLQIVNNTEGDWWLAHSLSTGQTGYIPSNYVAPSDS

>SUMO_Abl_SH3
MGSSHHHHHHGSGLVPRGSASMSDSEVNQEAKPEVKPEVKPETHINLKVSDGSSEIFFKIKKTTPLRRLM
EAFAKRQGKEMDSLRFLYDGIRIQADQTPEDLDMEDNDIIEAHREQIGGNDPNLFVALYDFVASGDNTLS
ITKGEKLRVLGYNHNGEWCEAQTKNGQGWVPSNYITPVNS

>SUMO_Src$_{Abl}$_SH3
MGSSHHHHHHGSGLVPRGSASMSDSEVNQEAKPEVKPEVKPETHINLKVSDGSSEIFFKIKKTTPLRRLM
EAFAKRQGKEMDSLRFLYDGIRIQADQTPEDLDMEDNDIIEAHREQIGGVTTFVALYDY**VASGDN**DLSFK
KGERLQIVNN**HNGE**WWLAHSLSTGQTGYIPSNYVAPSDS

>SUMO_Abl$_{Src}$_SH3
MGSSHHHHHHGSGLVPRGSASMSDSEVNQEAKPEVKPEVKPETHINLKVSDGSSEIFFKIKKTTPLRRLM
EAFAKRQGKEMDSLRFLYDGIRIQADQTPEDLDMEDNDIIEAHREQIGGNDPNLFVALYDF**ESRTET**TLS
ITKGEKLRVLGYN**TEGD**WCEAQTKNGQGWVPSNYITPVNS

>SUMO_W122C_Src_SH3
MGSSHHHHHHGSGLVPRGSASMSDSEVNQEAKPEVKPEVKPETHINLKVSDGSSEIFFKIKKTTPLRRLM
EAFAKRQGKEMDSLRFLYDGIRIQADQTPEDLDMEDNDIIEAHREQIGGVTTFVALYDYESRTETDLSFK
KGERLQIVNNTEGDW**C**LAHSLSTGQTGYIPSNYVAPSDS

>SUMO_W122C_Src$_{Abl}$_SH3
MGSSHHHHHHGSGLVPRGSASMSDSEVNQEAKPEVKPEVKPETHINLKVSDGSSEIFFKIKKTTPLRRLM
EAFAKRQGKEMDSLRFLYDGIRIQADQTPEDLDMEDNDIIEAHREQIGGVTTFVALYDY**VASGDN**DLSFK
KGERLQIVNN**HNGE**W**C**LAHSLSTGQTGYIPSNYVAPSDS

>SUMO_C100W_Abl_SH3
MGSSHHHHHHGSGLVPRGSASMSDSEVNQEAKPEVKPEVKPETHINLKVSDGSSEIFFKIKKTTPLRRLM
EAFAKRQGKEMDSLRFLYDGIRIQADQTPEDLDMEDNDIIEAHREQIGGNDPNLFVALYDFVASGDNTLS
ITKGEKLRVLGYNHNGEW**W**EAQTKNGQGWVPSNYITPVNS
